# Biomass production and nutrient use efficiency in white Guinea yam (*Dioscorea rotundata* Poir.) genotypes grown under contrasting soil mineral nutrient availability

**DOI:** 10.3389/fpls.2022.973388

**Published:** 2022-10-12

**Authors:** Ryo Matsumoto, Asrat Asfaw, Haruki Ishikawa, Kanako Takada, Hironobu Shiwachi, Robert Asiedu

**Affiliations:** ^1^ International Institute of Tropical Agriculture, Ibadan, Nigeria; ^2^ Tokyo University of Agriculture, Tokyo, Japan

**Keywords:** nutrient recovery efficiency, nutrient uptake, fertilizer response, low soil fertility, West Africa, genotypic variation

## Abstract

Yam (*Dioscorea* spp.) is of great importance to food security, especially in West Africa. However, the loss of soil fertility due to dwindling fallow lands with indigenous nutrient supply poses a challenge for yam cultivation. This study aimed to determine shoot and tuber biomass and nutrient use efficiency of white Guinea yam (*Dioscorea rotundata*) grown under low- and high-NPK conditions. Six white Guinea yam genotypes were used in field experiments conducted at Ibadan, Nigeria. Experiments were conducted with low soil NPK conditions with zero fertilizer input and high soil NPK conditions with mineral fertilizer input. Differences in response to soil NPK conditions, nutrient uptake, and nutrient use efficiency (apparent nutrient recovery efficiency) were observed among the tested genotypes. The genotypes TDr1499 and TDr1649, with high soil fertility susceptibility index (SFSI>1) and an increase in shoot and tuber biomass with fertilizer input, were recognized as susceptible to soil NPK conditions. There was a marked difference in apparent nutrient recovery efficiency; however, there was no varietal difference in physiological efficiency. Differences in apparent nutrient recovery efficiency among genotypes affected the fertilizer response (or susceptibility to soil NPK conditions) and the nutrient uptake. In contrast, the genotype TDr2029, with SFSI<1 and low reduction in shoot and tuber production between non-F and +F conditions, was recognized as a less susceptible genotype to soil NPK status. It was revealed that NPK fertilization did not reduce tuber dry matter content, regardless of genotype differences in susceptibility to soil NPK conditions. Hence, this could be helpful to farmers because it implies that yield can be increased without reducing tuber quality through a balanced application of soil nutrients. Our results highlight genotypic variation in sensitivity to the soil NPK availability, nutrient uptake, and nutrient use efficiency white Guinea yam. Differences in susceptibility to soil NPK conditions could be due to the genotypic variations in nutrient recovery efficiency white Guinea yam. Our findings could contribute to breeding programs for the development of improved white Guinea yam varieties that enhance productivity in low soil fertility conditions with low and high-input farming systems.

## Introduction

Yam (*Dioscorea* spp.) is a multispecies tuberous crop with immense potential for improving food security, especially with respect to the food and cultural systems of West Africa (Asiedu and Sartie, 2010); about 93% (66.8 million tons) of the global yam production occurs in this region (FAOSTAT, 2021). Among the species of *Dioscorea*, which vary in origin and distribution depending on tropical, subtropical, and temperate regions (Darkwa et al., 2020), white Guinea yam (*Dioscorea rotundata*) is predominantly cultivated and consumed in West Africa (Asfaw et al., 2020). The cultivation of white Guinea yam has steadily increased over the past few decades in West Africa, from 14.5 million tons in 1988 to 66.8 million tons in 2018 (FAOSTAT, 2021). The substantial increase in yam production has mainly been attributed to the expansion of the cultivation area rather than the productivity increase per unit area (FAOSTAT, 2021). The increase in yam productivity was marginal compared to that of potatoes (FAOSTAT, 2021).

In West Africa, yams are usually cultivated without chemical or organic fertilizers, often using landraces (Degras, 1993; Scott et al., 2000; Maliki et al., 2012). Traditionally, yam is the first crop after a long-term fallow because it requires fertile soils for optimum growth and yielding potential (Carsky et al., 2010). Diby et al. (2011a) reported that tuber yield was higher in fertile forest soils than in low-fertile savannah sites. It was suggested that soil fertility is crucial in yam cultivation. Similarly, Kassi et al. (2017) reported that soil organic carbon stocks contributed to the increased tuber yield, as *D. rotundata* crops harvested after *Chromoleana odorata* (green fertilizer) fallows produced the maximum yield. Consequently, yam producers perceive the decline in soil fertility as a critical constraint for yam production in areas under intensive cultivation (Lebot, 2019). Despite this, fertilizer use in Sub-Saharan Africa is generally low, partly because farmers do not recognize adequate profit opportunities with acceptable risks (Kaizzi et al., 2017).

The impact of fertilizer application on yam productivity remains unresolved due to several conflicting reports. While some studies have reported positive effects (Irving, 1956; Kpeglo et al., 1981; Lyonga, 1981; Diby et al., 2009; Diby et al., 2011c), others have reported no changes in that productivity (Kang and Wilson, 1981; Carsky et al., 2010). These discrepancies on the impact of fertilizer application on yam growth and yield could be attributed to the nutrient status of the experimental plots, as response to fertilization is affected by the soil fertility of the cultivation area. In the nutrient-poor savanna soils of Africa, the impact of fertilizer input on yam crops was positive, while it was significantly lower in the relatively fertile forest soils (Lugo et al., 1993; Diby et al., 2009; Diby et al., 2011c). Nevertheless, considering soil nutrient status, suitable fertilizer input can benefit crop yield. For example, several studies have reported that appropriate fertilizer application positively affected yam productivity in Sub-Saharan Africa (Lugo et al., 1993; Diby et al., 2009; Diby et al., 2011c; Cornet et al., 2022). Therefore, various soil management techniques are currently being developed, tested, and implemented to improve crop productivity in low-input farming systems in Africa (Kihara et al., 2020).

Matsumoto et al. (2021a) reported differential responses of white Guinea yam genotypes to available soil nutrients and identified genotypes with low soil nutrient tolerance and a high response to applied fertilizer. One of the factors for the difference in fertilizer response and tolerance to low-fertility soil among varieties is the difference in nutrient use efficiency (El-Sharkawy et al., 1998; Martí and Mills, 2002; Tamele et al., 2020). A better understanding of the physiological mechanism of fertilizer response and tolerance to low soil fertility is important for selecting and developing varieties suitable for cultivation under low fertilizer input and improving fertilizer utilization. However, there is little research on this aspect. Interspecific variation in nutrient uptake and nutrient use efficiency has been reported in *D. alata* and *D. rotundata* (Diby et al., 2011b; Hgaza et al., 2019); however, whether varietal differences exist in terms of nutrient uptake and nutrient use efficiency remains unknown. Although tuber dry matter content is a crucial characteristics highly valued by traders and consumers (Chukwu et al., 2007; Asiedu and Sartie, 2010), limited information is available regarding the effect of fertilizer on the percent dry matter content and its relationship with a fertilizer response of genotype. This study aimed to determine the biomass production and tuber dry matter content of different genotypes of white Guinea yam and their response to fertilizer input in terms of nutrient uptake and nutrient use efficiency. Our results would contribute to the development of cultivation techniques and varieties of white Guinea yam for improved biomass production and fertilizer response.

## Materials and methods

### Site and soil properties

**Figure 1:**
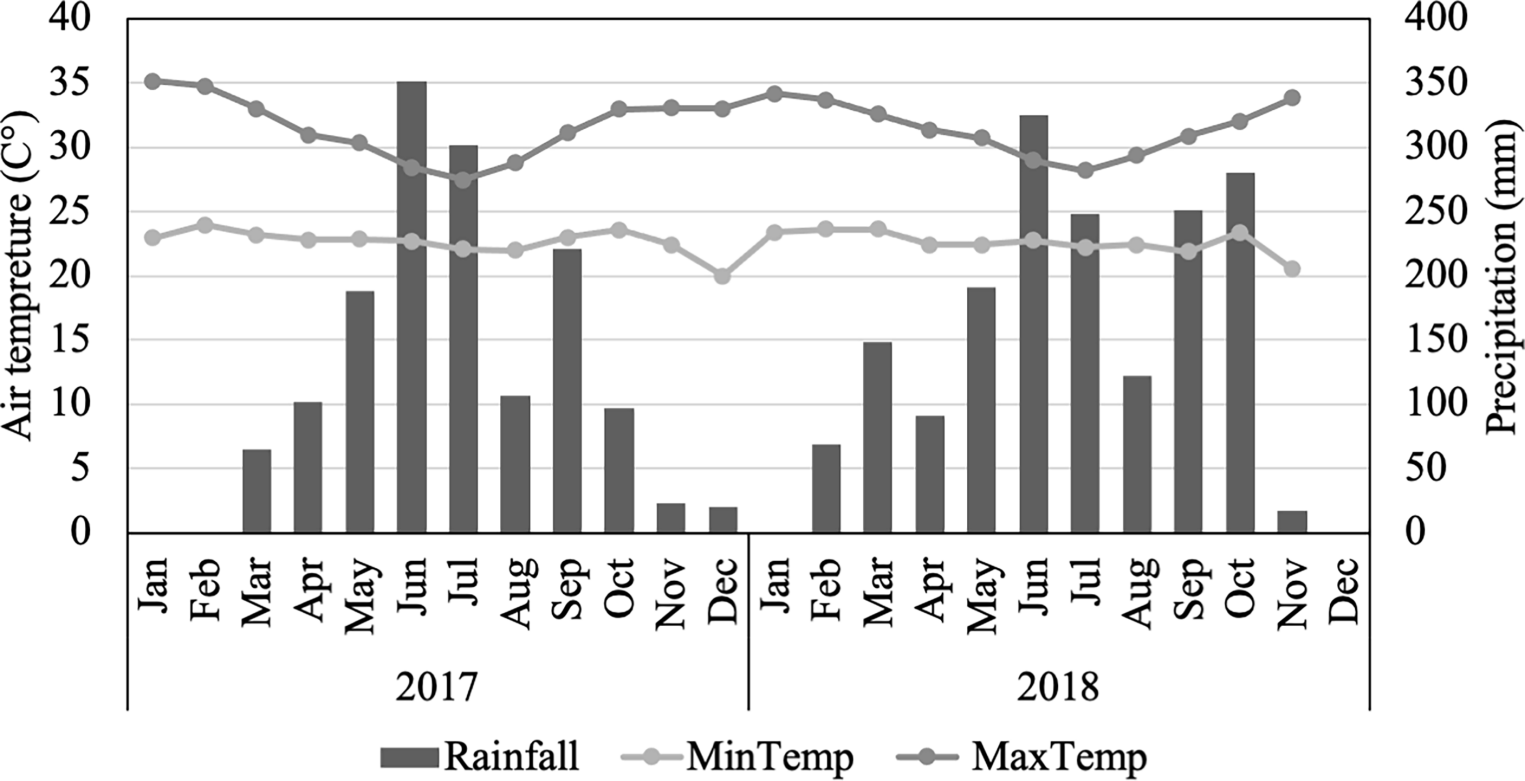
Air temperature and precipitation during the growth periods at the International Institute of Tropical Agriculture (IITA) Ibadan, Nigeria, in 2017 and 2018. Air temperature includes a black line and grey line representing maximum and minimum air temperature averages, respectively. For precipitation, data are cumulative values for every month.

Field experiments were conducted during the 2017 and 2018 cropping seasons (April to December) in the experimental field with low soil fertility at the International Institute of Tropical Agriculture (IITA), Ibadan, Nigeria (7° 29′ N, 3° 54′ E). The low soil fertility field was induced artificially by successive planting of cassava, maize, and sorghum, without fertilizer input in IITA, Ibadan (Matsumoto et al., 2021a). To assess the soil properties in the experimental field, soil samples were collected before conducting the experiment at depths of 0–20 cm from 30 randomly selected plots. Soil pH was determined by initially suspending the soil in water (1:2.5 soil:water ratio). Exchangeable Ca^2+^, Mg^2+^, K^+^, and available P were extracted according to the Mehlich-3 procedure (Mehlich, 1984). Cations were determined using an atomic absorption spectrophotometer (Accusys 211 Atomic Spectrophotometer, Buck Scientific, Connecticut, USA). P was assayed by colorimetric determination using a Genesys 10S UV-Vis spectrophotometer (Thermo Scientific, Waltham, MA, USA). Organic carbon was determined by chromic acid digestion with a spectrophotometric procedure using Genesys 10S UV-Vis spectrophotometer (Heanes, 1984). Total N was determined using the Kjeldahl method for digestion and colorimetric determination using a Technicon AAII Autoanalyzer (Seal Analytical, Wisconsin, USA) (Bremner and Mulvaney, 1982). Weather data for the experimental period were assessed using the data obtained from the Geographical Information System (GIS) unit of the IITA. **Figure 1** presents the meteorological conditions during the growth period, from planting to harvest (180 days after planting) for the trials. The total precipitation and average minimum/maximum temperatures for this period were 1410.5 mm, 22.7/30.7°C in 2017, and 1526.5 mm, 22.7/30.5°C in 2018, respectively.

### Plant materials and trial management

Field experiments were conducted using six genotypes of white Guinea yam. TDr1649 and TDr2484 were used in the 2017 field trial, while TDr1499, TDr1649, TDr1899, TDr2029, TDr2484, and TDr2948 were used for the 2018 trial. These genotypes are part of the mini-core collection of white Guinea yam (Pachakkil et al., 2021) maintained at the IITA. Those genotypes were selected based on the tuber yield and leaf density difference (**Table 1**). All the genotypes were multiplied under uniform conditions in the field at IITA headquarters during the 2016 and 2017 cropping seasons to generate high-quality planting material. Plants with symptoms of viral diseases, such as yam mosaic virus, were removed from the field during the growing period. Visually assessed clean tubers with no signs of rot or pests were used as seed tuber materials for the trials. Tubers weighing approximately 1–2 kg were cut horizontally to remove the head and tail components. The tuber centre component was cut into 50 ± 10 g pieces to obtain uniform material for planting (yam setts). Yam setts were treated with a mixture of 70 g mancozeb (fungicide) and 75 mL chlorpyrifos (insecticide) dissolved in a 10 L volume of tap water for 5 min, and the setts were dried for 20 h in the shade before planting for pre-sprouting. The yam setts were planted in plastic pots (12 cm diameter × 10 cm height) filled with topsoil on 4 May 2017 and 2 May 2018. Because variation in the sprout emergence time was the main cause of variation in shoot biomass size and tuber yield within plots in yam trials (Cornet et al., 2014), seedlings that germinated simultaneously (within 14 days difference in sprout emergence date) were selected and used as experimental material in this study. Plants with uniform sprouts were transplanted into the field in a 0.5 m × 1.0 m arrangement to give 20,000 plants per hectare. A 2 m stake was provided for each plant at 30 days after planting.

**Table 1:** Variation in tuber yield (g plant^-1^) and leaf density in the white Guinea yam (*Dioscorea rotundata*) mini-core collection and the distribution of the used genotypes.

	Leaf density	Fresh tuber yield (g plant^-1^)
*Mini-core collection (n=102)*
Mean	1.9	1234.9
Standard deviation	0.5	493.0
Coefficient of variance (%)	26.5	39.9
*Genotype selected from mini-core collection*
TDr1499	2.7a	2726.0a
TDr1649	2.1b	2107.7ab
TDr1899	2.0b	1456.3b
TDr2029	2.0b	1401.0b
TDr2484	2.0b	2065.3ab
TDr2948	2.0b	1562.7b

The leaf density of plants was rated on a scale of 1 to 3 where 1= low, 2 = intermediate, and 3= high. Leaf density was recorded based on yam descriptors approximately 100 days after germination as the middle growth stage. Each point represents the mean of six data points. Different letters in the same column indicate signiﬁcant differences (p<0.05) as determined by Tukey’s HSD test. Data were obtained from a field evaluation at the International Institute of Tropical Agriculture (IITA) in 2014 (Pachakkil et al. (2021).

The plants were transplanted into the field on a ridge approximately 40 cm high and 60 cm wide. The field experiment was laid out in a split-plot randomized block design with four replications. The main plot comprised two levels of fertilizer treatments, non-fertilized (Non-F) and fertilized (+F), while the subplot consisted of genotypes. The size of the subplot was 15 m^2^. The fertilized plot (+F) received 90, 50, and 75 kg nitrogen (N), phosphorus (P), and potassium (K) per hectare, respectively. These were based on the recommendations of fertilizer amounts for low soil fertility conditions (Chude et al., 2012). In the 2017 trial, the total number of plots was 16 (two soil nutrient fertility levels × 2 genotypes × 4 replications) (detailed field design presented in **Supplementary Material 1**). In the 2018 trial, the total number of plots was 48 (two soil nutrient fertility levels × 6 genotypes × 4 replications) (detailed field design presented in **Supplementary Material 2** **)**. Fertilizer was applied 14 days after transplanting using the side dressing method in both the 2017 and 2018 trials. To avoid fertilizer contamination, there was a 20 m distance between the non-fertilized and fertilized plots. Weeds were manually removed whenever present to maintain weed-free plots throughout the experiment.

### Evaluation of shoot and tuber productivity under different soil fertility

At 180 days after planting, three plants from each subplot were selected randomly, excluding border plants in both the 2017 and 2018 trials, to analyse the effect of soil NPK conditions on shoot and tuber production. The total number of harvested samples was 12 (three plants × 4 replicates) for each genotype in both the 2017 and 2018 trials. Harvested plants were separated into leaves, stems, and tubers. Plant parts were rinsed with tap water. The total weight of fresh tuber was recorded. All leaves, stems, and a weighed sample of the tubers were dried in an oven at 80°C for three days. Dried samples were weighed to determine the total leaf and stem dry weights. The percent dry matter content of the tuber was calculated from the dry weight of the tuber sample in relation to its fresh weight. The total tuber dry weight was determined by multiplying the total fresh tuber weight by the percent dry matter content of the tuber. The percentage difference in dry shoot weight and dry tuber weight due to the difference in NPK conditions was calculated using the following formula:


$$Percentage\;difference\;\left(\%\right)=\left(\frac{\bar{xhf}-\bar{xlf}}{\bar{xhf}}\right)\times \;100$$

where $\bar{xlf}$ and $\bar{xhf}$ are the mean trait values of a given genotype in non-F and +F environments, respectively.

Soil fertility susceptibility index (SFSI) was calculated using the following formula **(** Fischer and Maurer, 1978):


$$Soil\;fertility\;susceptibility\;index\;\left(SFSI\right)=\left(1-\frac{\bar{xlf}}{\bar{xhf}}\right)/\left(1-\frac{\bar{Ylf}}{\bar{Yhf}}\right)$$

where $\bar{xlf}$ and $\bar{xhf}$ are the mean trait values of a given genotype under the non-F and +F conditions, respectively. $\bar{Ylf}$ and $\bar{Yhf}$ are the mean trait values of all genotypes under the non-F and +F conditions, respectively, and $1-\bar{Ylf}/\bar{Yhf}$ is the soil fertility intensity index.

### Nutrient uptake and use efficiency

Dried leaf and tuber samples from the 2018 trial were used to determine the nutrient content in plants. Dried leaf and tuber tissues were ground separately in a Wiley mill and passed through a ≤1 mm mesh screen. As per pre-standard methods, an NC analyser (Sumigraph NCH-22, Sumika Chemical Analysis Service Ltd., Japan) was used to determine the nitrogen content in leaf and tuber tissues (Anderson and Ingram, 1993). Ground dry plant samples (100 mg) samples were pyrolyzed with 5 mL of nitric acid, and the filtered samples were analysed to determine the P and K content in leaves and tubers using inductively coupled plasma optical emission spectrometry (ICP-OES; iCAP 6000 Series, Thermo Fisher Scientific, MA, United States). The N, P, and K uptakes by the plants was determined by adding the product of the dry weight of each plant part with the elemental concentration of each plant part. In the current study, the following fertilizer efficiency parameters were used (Craswell and Godwin, 1984):


$$\text{Apparent nutrient recovery efficiency}=\left({\text{U}}_{\text{tF}}-{\text{U}}_{\text{tN}}\right)/F x 100$$

$$\text{Physiological efficiency}=\left({\text{T}}_{\text{wF}}-{\text{T}}_{\text{wN}}\right)/\left({\text{U}}_{\text{tF}}-{\text{U}}_{\text{tN}}\right)$$

where *U_tF_* is total nutrient uptake in plants under +F condition. *U_tN_* is total nutrient uptake in plants under non-F condition; F is nutrient supply (g/plant); *T_wF_* is dry tuber weight under +F condition; *T_wN_* is dry tuber weight under non-F condition. Apparent nutrient recovery efficiency was calculated as the efficiency of nutrient capture from soil and/or fertilizer input. Physiological efficiency was calculated as the efficiency of capturing plant nutrients in tuber yield.

### Statistical analysis

Data were analysed using the linear mixed model in the lme4 package (Bates, 2010) in the R environment version 4.0.3 for statistical computing (R Core Team, 2018). To determine a significant difference between the mean values of traits obtained from non-F and +F conditions for each genotype, t-test was performed using the R package ggpubr (Kassambara, 2020). Multiple comparison analysis using Tukey’s HSD test was performed to detect statistically significant differences in the obtained traits among varieties using the agricolae package (de Mendiburu, 2021). Correlation analysis among the tested parameters was determined using Pearson correlation coefficients. In all calculations, statistical significance was set at p < 0.05.

## Results

### Soil properties at the experimental sites in 2017 and 2018

The soil chemical properties of the experimental fields are presented in **Table 2**. Soil pH was 5.69 in 2017 and 5.98 in 2018. The organic carbon content was 0.24% and 0.39% in 2017 and 2018, respectively. No total N content change was observed between the 2017 and 2018 trials (0.04%). The available P content in 2017 (1.18 mg kg^−1^) was lower than that in the 2018 trial (2.21 mg kg^−1^). Exchangeable Ca and Mg in 2017 were higher than in those in the 2018 trial. Exchangeable K was 0.20 cmol[+] kg^−1^ in 2017, which was higher than that in 2018 (0.08 cmol[+] kg^−1^).

**Table 2:** Soil chemical properties of the experimental site at IITA Ibadan, Nigeria.

Soil chemical properties	2017		2018	
	Mean	SD (n=6)	Mean	SD (n=6)
pH	5.69	0.21	5.98	0.04
Organic carbon (%)	0.24	0.04	0.39	0.11
Total nitrogen (%)	0.04	0.02	0.04	0.00
Available phosphorus (mg kg^-1^)	1.18	0.82	2.21	0.91
Calcium (cmol[+] kg^-1^)	2.90	0.46	0.75	0.14
Magnesium (cmol[+] kg^-1^)	0.69	0.11	0.23	0.11
Potassium (cmol[+] kg^-1^)	0.20	0.07	0.08	0.01

SD, standard deviation.

### Effect of fertilizer treatment on shoot and tuber production

**Figure 2:**
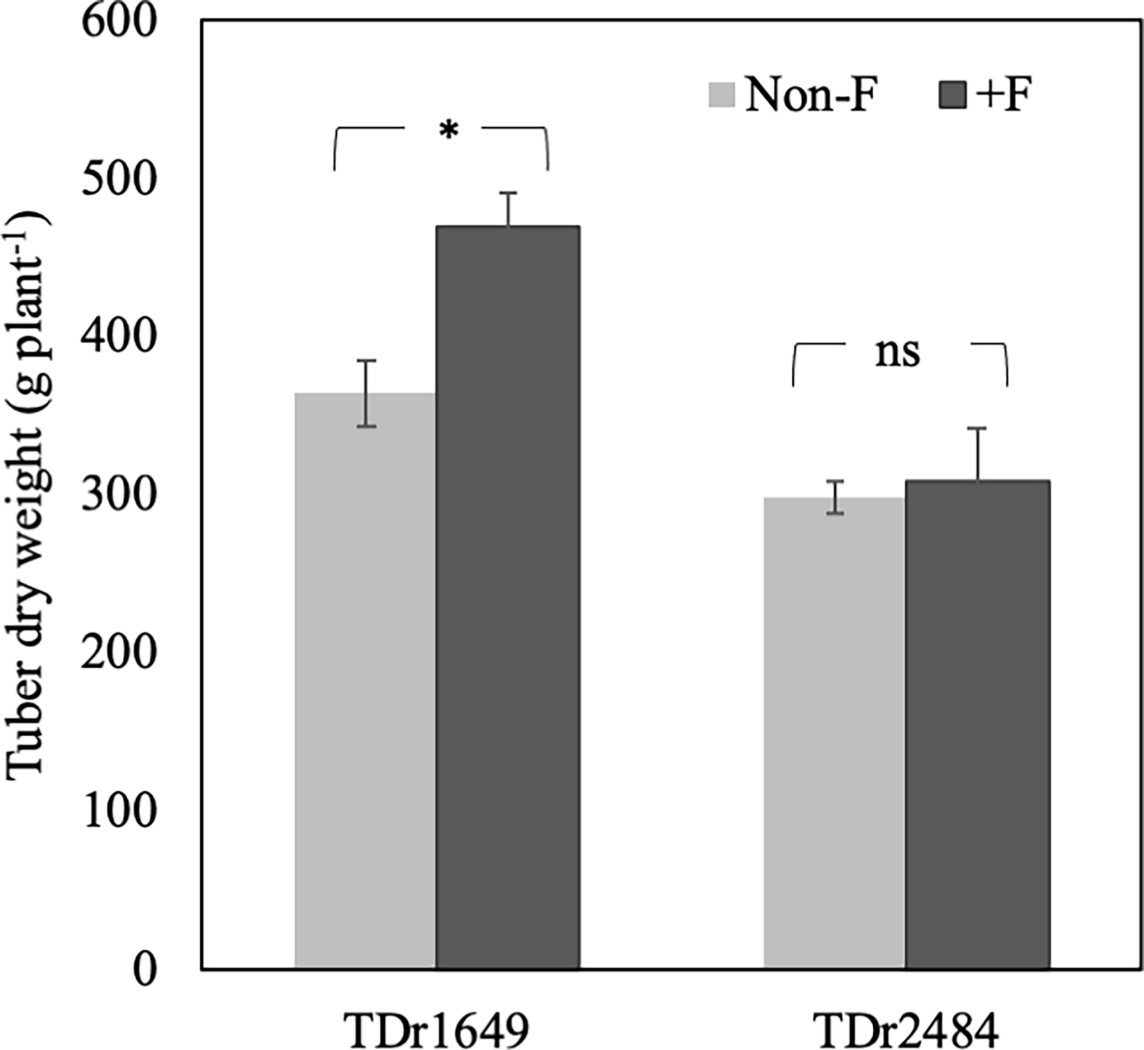
Effect of fertilizer treatment on dry tuber weight (g plant^-1^) in two white Guinea yam (*Dioscorea rotundata*) genotypes in 2017. * represents a significant difference at *p*< 0.05 calculated by t-test between non-fertilized (non-F) and fertilized (+F) conditions. ns; significant difference at p < 0.05 calculated by t-test between non-fertilized (non-F) and fertilized (+F) conditions.

The effect of fertilizer application on dry tuber weight in the 2017 trial is presented in **Figure 2**. Fertilizer application increased the dry tuber weight of TDr1649 plants in 2017 experimental trial. However, there was no significant difference in the dry tuber weight of TDr2484 grown between the non-F and +F conditions.

In the 2018 trial, genotype and fertilizer application interactions were significant for the dry shoot weight (**Table 3** and **Supplementary Material 3**). Although the difference was not statistically significant, the shoot dry weight of TDr1499 was the highest among the tested genotypes. However, there was a significant difference in the dry shoot weight among the tested genotypes under +F condition, and the highest dry shoot weight was observed in TDr1499 (**Table 3** **)**. The percent difference in dry shoot weight due to non-fertilizer application ranged from 14.3% (TDr2029) to 41.1% (TDr1499). SFSI for dry shoot weight ranged from 0.46 to 1.31. Among the genotypes tested under the two different fertilizer treatments, TDr1499 and TDr1649 produced significantly higher dry shoot weights in the +F condition than in the non-F state (**Table 3** **)**.

**Table 3:** Effect of genotype and fertilizer treatment on the shoot and tuber weight in six white Guinea yam (*Dioscorea rotundata*) genotypes In the 2018 trial.

	Dry shoot weight (g plant^-1^)						Dry tuber weight (g plant^-1^)					
	Non-F	+F	Mean	PD	SFSI	p-value	Non-F	+F	Mean	PD	SFSI	p-value
TDr1499	76.9a	130.5a	103.7a	41.1	1.31	0.00	235.5a	489.9a	362.7a	51.9	1.44	0.00
TDr1649	64.5a	108.1ab	86.3ab	40.3	1.28	0.01	235.8a	433.3ab	334.5ab	45.6	1.26	0.01
TDr1899	45.3a	60.7c	53.0bc	25.5	0.81	0.29	155.8a	239.0d	197.4b	34.8	0.96	0.10
TDr2029	48.3a	56.4c	52.4c	14.3	0.46	0.60	260.0a	313.8bcd	286.9ab	17.2	0.48	0.41
TDr2484	48.6a	63.7c	56.2bc	23.7	0.75	0.12	200.0a	262.5cd	231.2ab	23.8	0.66	0.09
TDr2948	57.8a	78.3bc	68.1bc	26.2	0.83	0.14	260.7a	370.3bcd	315.5ab	29.6	0.82	0.08
Mean	56.9	83.0					224.6		351.5			
Type II Wald chi-square tests (Chisq)												
Genotype (G)	30.7***						69.0***					
Treatment (T)	66.2***						23.6***					
Interaction G × T	12.4*						13.0*					

Non-F, non-fertilized; +F, fertilized. PD, percent difference due to contrasting soil mineral nutrient conditions. SFSI, soil fertility susceptibility index. Different letters in the same column indicate signiﬁcant difference (p<0.05) as determined by Tukey’s HSD test. ***p < 0.001, *p < 0.05, analysed by t-test between -F and +F conditions.

Although dry tuber weight in non-F conditions ranged from 155.8 g plant^-1^ (TDr1899) to 260.7 g plant^-1^ (TDr2948), no significant difference was observed among the tested genotypes. Under the +F conditions, TDr1499 had the highest dry tuber weight (489.9 g plant^-1^), while TDr1899 had the lowest dry tuber weight (239.0 g plant^-1^) among the tested genotypes. Genotype TDr1499 showed a 51.9% reduction in dry tuber weight in the non-F condition compared to that in the +F condition. The lowest reduction in dry tuber weight of 17.2% was recorded for the genotype TDr2029. The SFSI for dry tuber weight ranged from 0.48 to 1.44. Among the genotypes tested under the two different fertilizer conditions, TDr1499 and TDr1649 produced significantly higher dry tuber weights in the +F condition than in the non-F state (**Table 3** and **Supplementary Material 3** **)**.

Genotype was significant for the percent dry matter content of the tuber **(** **Table 4** **).** The effect of genotype and fertilizer treatment interaction on tuber percent dry matter content was not observed (**Table 4**). Fertilizer application did not increase the percent dry matter content of the tuber in all genotypes. Under the non-F conditions, TDr1649 (31.2%), TDr1899 (32.5%), and TDr2029 (31.0%) had a significantly higher dry matter content of tuber than TDr2428 (25.5%). TDr1649 and TDr1899 showed higher percent dry matter content of tuber than TDr1499, TDr2029, TDr2484, and TDr2048 under +F conditions (**Table 4**).

**Table 4:** Effect of genotype and fertilizer treatment on percent dry matter content of tuber (%) in six white Guinea yam (*Dioscorea rotundata*) genotypes in the 2018 trial.

	Non-F	+F	p-value
TDr1499	27.8ab	28.6b	0.37
TDr1649	31.2a	34.1a	0.22
TDr1899	32.5a	33.1a	0.39
TDr2029	31.0a	29.3b	0.56
TDr2484	25.5b	27.0b	0.49
TDr2948	28.2ab	28.7b	0.51
Mean	29.37	30.13	
Type II Wald chi square tests (Chisq)			
Genotype (G)	94.2***		
Treatment (T)	0.7		
Interaction G x T	7.5		

Non-F, non-fertilized; +F, fertilized. Different letters in the same column indicate signiﬁcant difference (p<0.05) as determined by Tukey’s HSD test. p-value was calculated using t-test between -F and +F conditions. ***p < 0.001, analysed by t-test between -F and +F conditions.

### Nutrient uptake

Fertilizer treatment increased the N and K uptake of TDr1499 and TDr1649. The interaction between genotype and fertilizer treatment was significant for P uptake (*p<* 0.001) (**Table 5**). The difference in genotype did not affect P uptake under non-F conditions. However, P uptake varied with genotype differences under +F conditions (**Table 5**). The genotypes TDr1499, TDr1649, and TDr2948 accumulated higher P than TDr1899, TDr2029, and TDr2484 under +F conditions. N uptake (g plant^-1^) by the genotypes was 7.0 to 9.7 times higher than that of P under non-F conditions, whereas N uptake was 5.8 to 7.7 times higher than that of P under the +F conditions. Similarly, the K uptake (g plant^-1^) was 5.7 to 9.5 times higher in the non-F condition compared to that of P, which was 4.7 to 6.3 times higher in the +F condition (**Table 5**).

**Table 5:** Effect of genotype and fertilizer treatment on the uptake of nitrogen, phosphorus, and potassium in six white Guinea yam (*Dioscorea rotundata*) genotypes in the 2018

	Nitrogen uptake(g plant^-1^)			Phosphorus uptake(g plant^-1^)			Potassium uptake(g plant^-1^)		
	Non-F	+F	p-value	Non-F	+F	p-value	Non-F	+F	p-value
TDr1499	2.6a	4.7a	0.00	0.3a	0.8a	0.00	2.2a	3.9a	0.01
TDr1649	2.2a	3.7ab	0.01	0.3a	0.6a	0.00	2.0a	2.8b	0.10
TDr1899	1.5a	2.3c	0.11	0.2a	0.3b	0.14	1.4a	1.9b	0.16
TDr2029	2.1a	2.9bc	0.26	0.3a	0.4b	0.12	1.7a	2.1b	0.48
TDr2484	1.9a	2.6bc	0.07	0.2a	0.4b	0.07	1.7a	2.1b	0.29
TDr2948	2.9a	3.5ab	0.43	0.3a	0.6a	0.01	2.1a	2.9ab	0.13
Mean	2.2	3.3		0.3	0.5		1.9		2.6
Type II Wald chi-square tests (Chisq)									
Genotype (G)	38.4***			66.8***			33.9***		
Treatment (T)	31.2***			87.8***			17.1***		
Interaction G x T	8.6			28.4***			9.9		

Non-F, non-fertilized; +F, fertilized. Different letters in the same column indicate signiﬁcant differences difference (p<0.05) as determined by Tukey’s HSD test. ***p < 0.001, analysed by t-test between -F and +F conditions.

### Nutrient use efficiency parameters

**Figure 3:**
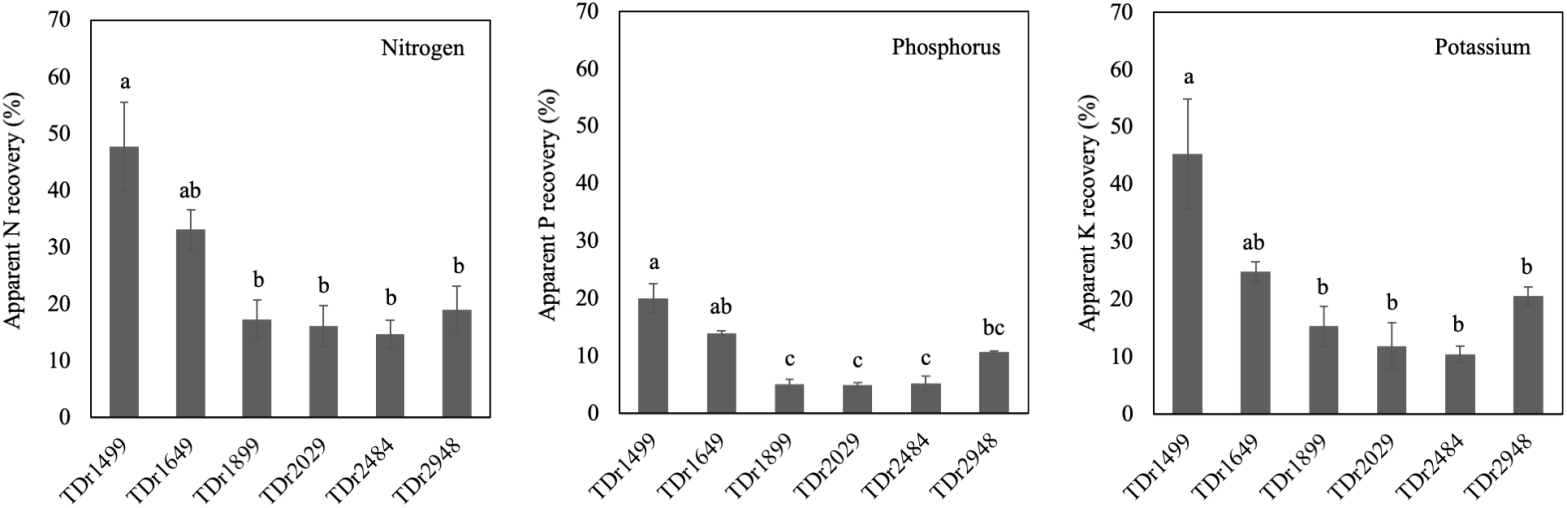
Apparent nutrient recovery efficiency of six genotypes in white Guinea yam (*Dioscorea rotundata*). Nitrogen (left box), phosphorus (centre box), and potassium physiological efficiency (right box) are shown. All values are expressed as means. Different alphabet indicates statistical significance (*P*< 0.05). Bars represent presents a standard error.

Apparent nutrient recovery efficiency and physiological efficiency that already include the response to soil nutrient level were estimated considering varietal differences. Varietal difference in the apparent nutrient recovery efficiency was observed among the tested genotypes (**Figure 3**). Nitrogen apparent nutrient recovery efficiency ranged from 14.7 (TDr2484) to 47.7% (TDr1499). Nitrogen apparent nutrient recovery efficiency of TDr1499 was significantly higher than that of TDr1899, TDr2029, TDr2484, and TDr2948. Similar results were observed with apparent nutrient recovery efficiency for phosphorus and potassium.

Nitrogen physiological efficiency did not differ among the tested genotypes (p = 0.141). Similar results were observed in potassium physiological efficiency (*p* = 0.780). Phosphorus physiological efficiency showed high values, which ranging from 462.2 to 690.7 g plant^-1^; however, varietal difference was not observed significantly among the tested genotypes (*p* = 0.208).

### Correlation analysis among tested parameters (dry tuber weight and nutrient uptake)

**Figure 4:**
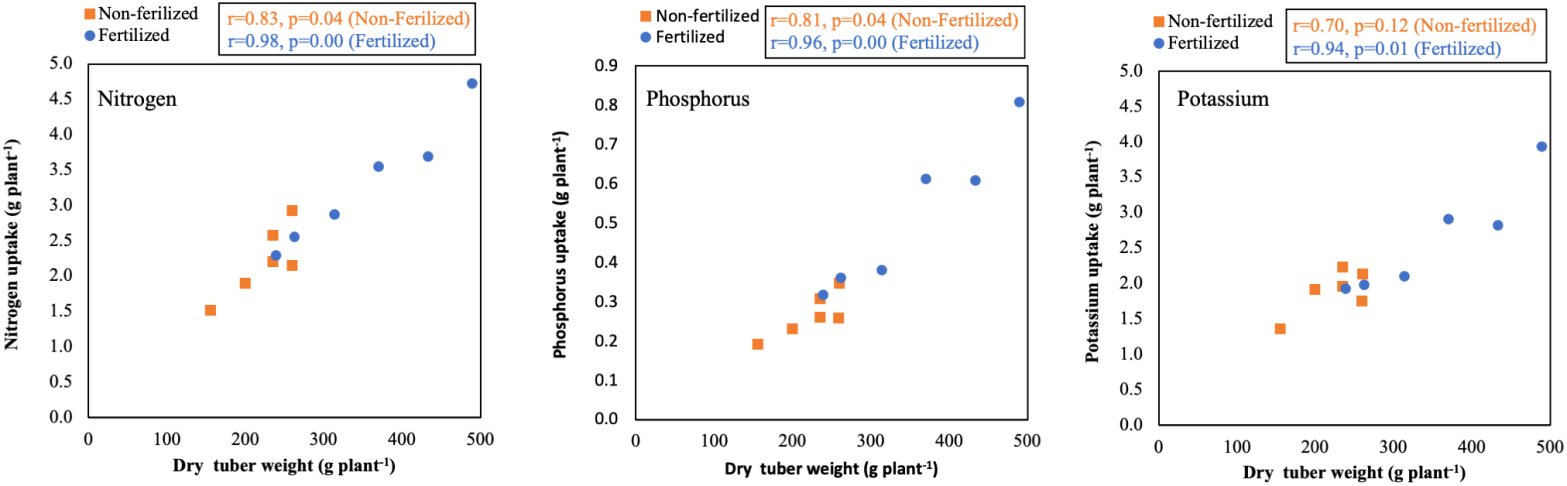
Correlation between dry tuber weight and nutrient uptake under non-fertilized and fertilized conditions in six white Guinea yam (*Dioscorea rotundata*) genotypes. Relationships for nitrogen (left box), phosphorus (centre box), and potassium (right box) uptake are shown.

The correlation between dry tuber weight and nutrient uptake is presented in **Figure 4**. The dry tuber weight and N uptake were strongly and positively correlated in the non-F and the +F groups, respectively: r = 0.83 (*p* = 0.04) and 0.98 (*p* = 0.00). A similar trend was obtained between the relationship between the P and K uptake and dry tuber weight.

## Discussion

Soil fertility in Nigeria has been categorized into five levels (Chude et al., 2012). Although the critical soil nutrient levels for yam cultivation have not yet been established in West Africa, yam cultivation in soils containing< 0.1% N,< 10 mg kg^−1^ available P, and 0.15 Cmol[+] kg ^−1^ of exchangeable K requires external fertilizer inputs (Carsky et al., 2010). According to soil analysis performed before the experiments and previous reports by Chude et al. (2012) and Carsky et al. (2010), our experimental field in both the 2017 and 2018 trials had low soil NPK availability and could be inferred as infertile to sustain normal yam plant growth and optimize the yield (**Table 2**). It is, therefore, more likely to observe the positive effect of added fertilizer input in the growth and yield of yam under low soil fertility conditions. However, the fertilizer response to biomass production and tuber yield varied among the genotypes studied, suggesting that the response of soil NPK levels or fertilizer input in white Guinea yam could be genotype-specific (**Figure 2**, **Table 3**, and **Supplementary Material 3**).

Our results indicated differences among the white Guinea yam genotypes in the nutrient uptake **(** **Table 5**). The studied white Guinea yam genotypes absorbed N and K as primary nutrients during growth and showed an increase in tuber yield with an increase in nutrient uptake (**Figure 4**); this is also consistent with the results presented by Diby et al. (2011b) and Irizarry et al. (1995). The genotypes TDr1499 and TDr1649, with high response to fertilizer input, showed higher nutrient uptake than the other genotypes. In other words, the genotypes depended on the high soil fertility to exhibit high productivity. Thus, the genotypes TDr1499 and TDr1649, of Togolese origin, were responsive to fertilizer input and could be suitable candidates to maximize productivity under a high-input cultivation system.

The effect of fertilizer application on yam tuber yield is variable and sometimes conflicting (Irving, 1956; Kang and Wilson, 1981; Kpeglo et al., 1981; Lyonga, 1981; Diby et al., 2009; Carsky et al., 2010; Diby et al., 2011c). Diby et al. (2009) and Carsky et al. (2010) discussed differences in the reports on the beneficial impact of fertilizer application on yam growth and yield could be attributed to the nutrient status of the cultivation area. Dare et al., (2010, 2013, 2014) reported the potential influence of arbuscular mycorrhizal fungi that occur naturally in the yam growing areas in some of the observed variability.

This study revealed, for the first time, that there are varietal differences in the apparent recovery efficiency of white Guinea yam (**Figure 3**). Hgaza et al. (2019) also reported interspecific variations between the *D. alata* and *D. rotundata* genotypes in nitrogen uptake and apparent nitrogen recovery efficiency (total nitrogen uptake from fertilizer/total nitrogen rate applied × 100). Nutrient uptake in studied white Guinea yam genotypes increased with an apparent increase in nutrient recovery efficiency under +F conditions but not in physiological nutrient efficiency. This suggests the difference in nutrient recovery efficiency affected the fertilizer response or susceptibility to soil NPK condition in the white Guinea yam.

Species or cultivars with a high growth rate usually respond more favorably to fertilizer application than those with low growth rates (Mengel, 1983). Our result corroborates the findings of Mengel (1983). The fertilizer-responsive genotypes, TDr1499 and TDr1649, produced more vigorous shoot biomass than the other genotypes, which did not respond to applied fertilizer **(** **Table 3**). Therefore, the size of shoot biomass or leaf density as a morphological traits, associated with plant demand for nutrients, might be a factor contributing to varietal difference in fertilizer or soil NPK responsiveness among the tested genotypes.

Iseki et al. (2022) reported a positive correlation between shoot biomass and tuber yield and pointed out that shoot growth is important for final tuber yield. Therefore, increasing shoot growth by fertilizer application may improve tuber yield in white Guinea yam genotypes with a high response to fertilizer input. TDr1499 and TDr1649 were responsive to the fertilizer input with increased nutrient uptake, indicating that they can exhibit high productivity under high nutrient input. Productivity improvement in white Guinea yam in West Africa could be expected by combining appropriate fertilization techniques (Cornet et al., 2022) and genotypes that respond well to fertilizer under a high input system.

In contrast to TDr1499 and TDr1649, the genotypes TDr1899, TDr2029, TDr2484, and TDr2948 showed SFSI<1 and a reduction in shoot and tuber production from +F to non-F conditions (**Table 3**). These results indicate that these genotypes were tolerant to low soil NPK conditions or less susceptible to soil NPK status. Among the genotypes, TDr2029 was the least sensitive to soil NPK conditions. The genotypes with a high and stable yield of marketable tubers are selection targets for breeding programs for yam (Otoo et al., 2006; Asiedu and Sartie, 2010). Hence, TDr2029, of Nigerian origin, could be a potential parent to generate varieties with a stable yield and less sensitive to soil fertility conditions or the best candidate for immediate release as a new variety for the low input system in West Africa.

Likewise the tuber yield, the percent dry matter content of the tuber also varied among genotypes in white Guinea yam (**Table 4**). This result was consistent with Matsumoto et al. (2021b), who investigated the genotype × environment interaction on tuber quality traits on white Guinea yam. However, the application of NPK fertilizer did not affect the percent tuber dry matter content, regardless of the genotype differences in susceptibility to soil NPK conditions. This means the application of NPK increased fresh tuber yield and hence dry tuber yield without significantly influencing percent dry matter content. This is helpful to farmers because it implies that yield can be increased without reducing tuber quality by using a balanced application of soil nutrients. This is contrary to the fears expressed by some farmers that fertilizer application will reduce yam tuber quality. Our result is in line with Gizachew et al. (2022), who reported that neither organic nor mineral fertilizer application affects cassava tuber quality.

In addition to fertilizer application and soil amendments, the use of cultivars with high nutrient use efficiency and responsiveness to external nutrient supply would improve productivity in the yam cultivation system under low soil fertility conditions (Asiedu and Sartie, 2010). Breeding efforts should, therefore, focus on attributes such as high yield and high nutrient use efficiency in yams. Our results highlight the genotypic variations in white Guinea yam with respect to susceptibility to soil NPK availability, nutrient uptake, and nutrient use efficiency. The wide diversity of fertilizer response or non-susceptibility to soil NPK status might be expected in the mini-core collection of white Guinea yam, confirming the wide range of tuber yield and leaf density (Pachakkil et al., 2021). The contrasting genotypes with unique characteristics of high and less susceptibility to soil nutrient conditions provide a good opportunity for further studies to elucidate the genetic and physiological bases for and the influence of genotype × environment (including soil microbes) interactions on the differential response to these major nutrients in yam. Our findings also serve as reference for breeding new and improved varieties for low and high-input cultivation systems in West Africa.

## Data availability statement

The raw data supporting the conclusions of this article will be made available by the authors, without undue reservation.

## Author contributions

Conceptualization, RM; methodology and formal analysis, RM and KT; investigation and fieldwork, RM and KT; writing-original draft preparation, RM; writing-review and editing, KT, HI, HS, AA and RA; project administration, HI; funding acquisition, HI. All authors contributed to the article and approved the submitted version.

## Funding

This research was supported by the Ministry of Agriculture, Forestry, and Fisheries of Japan through the “MAFF yam project”.

## Acknowledgments

The authors wish to thank the International Institute of Tropical Agriculture (IITA) for allowing us to use their facilities. We acknowledge the IITA staff for their support. Part of the data used in this study was generated under the project “Use of genomic information and molecular tools for yam germplasm utilization and improvement for West Africa (EDITS-Yam; 2011-2015)” of the Japan International Research Center for Agricultural Science, in collaboration with the Iwate Biotechnology Research Center and the IITA.

## Conflict of interest

The authors declare that the research was conducted in the absence of any commercial or financial relationships that could be construed as a potential conflict of interest.

## Publisher’s note

All claims expressed in this article are solely those of the authors and do not necessarily represent those of their affiliated organizations, or those of the publisher, the editors and the reviewers. Any product that may be evaluated in this article, or claim that may be made by its manufacturer, is not guaranteed or endorsed by the publisher.
