# Supplementary figures and images for: Biomass production and nutrient use efficiency in white Guinea yam (Dioscorea rotundata Poir.) genotypes grown under contrasting soil mineral nutrient availability
Source: Front Plant Sci. 2022 Oct 12;13:973388. doi: 10.3389/fpls.2022.973388 (PMC9597467; doi:10.3389/fpls.2022.973388)

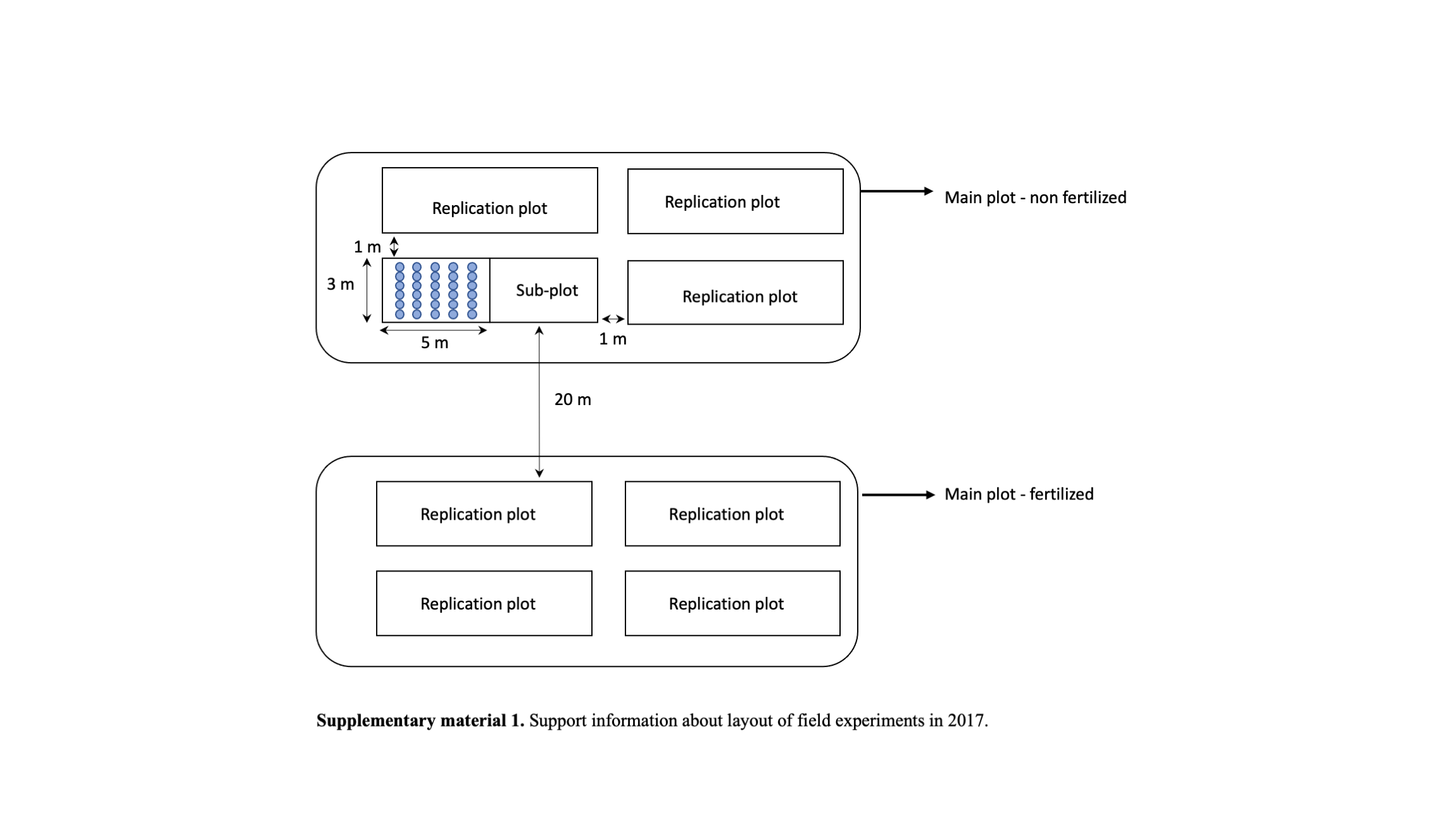

Supplement: Supplementary file 1 [file Image_1.tiff]

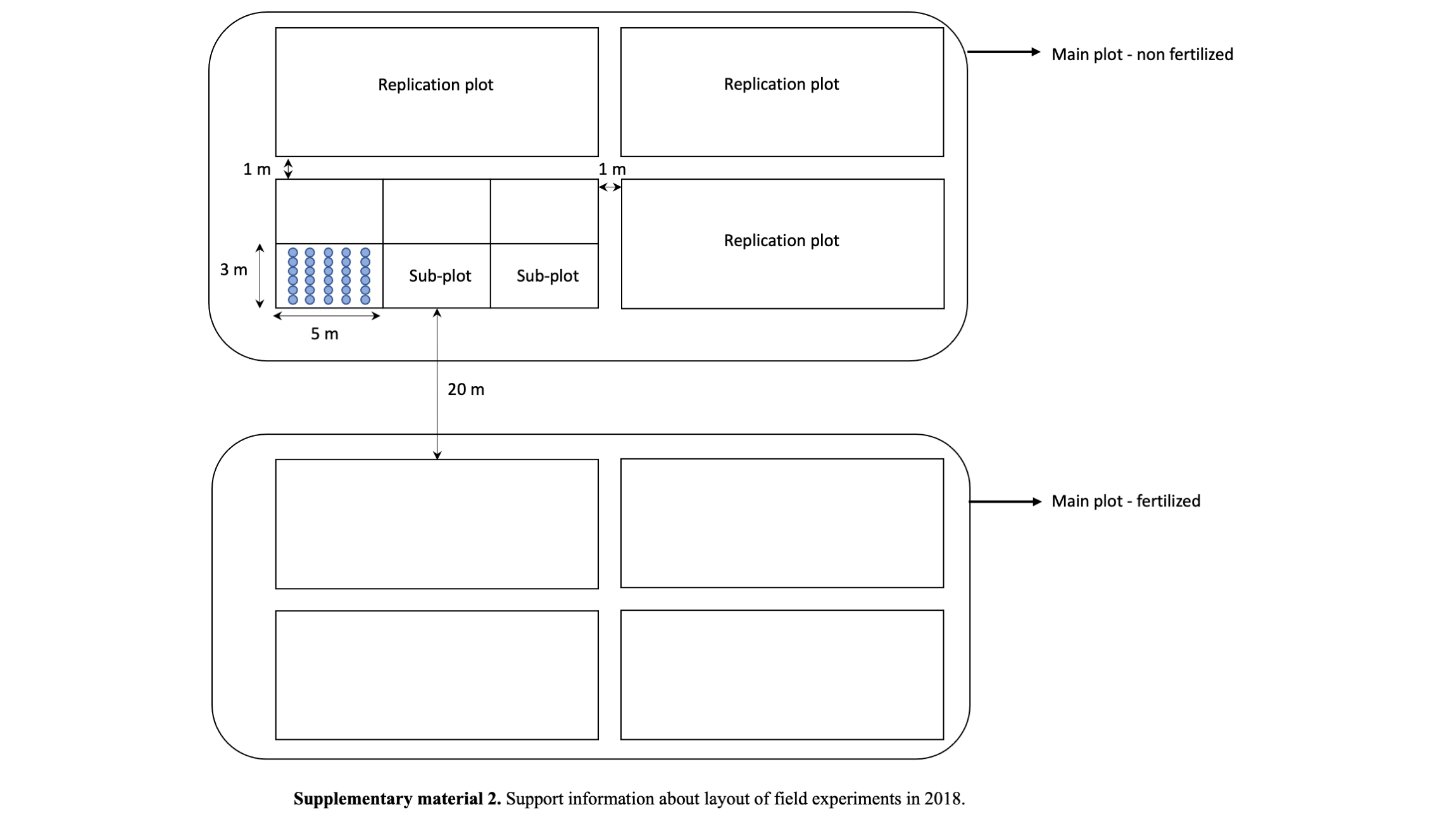

Supplement: Supplementary file 2 [file Image_2.tiff]

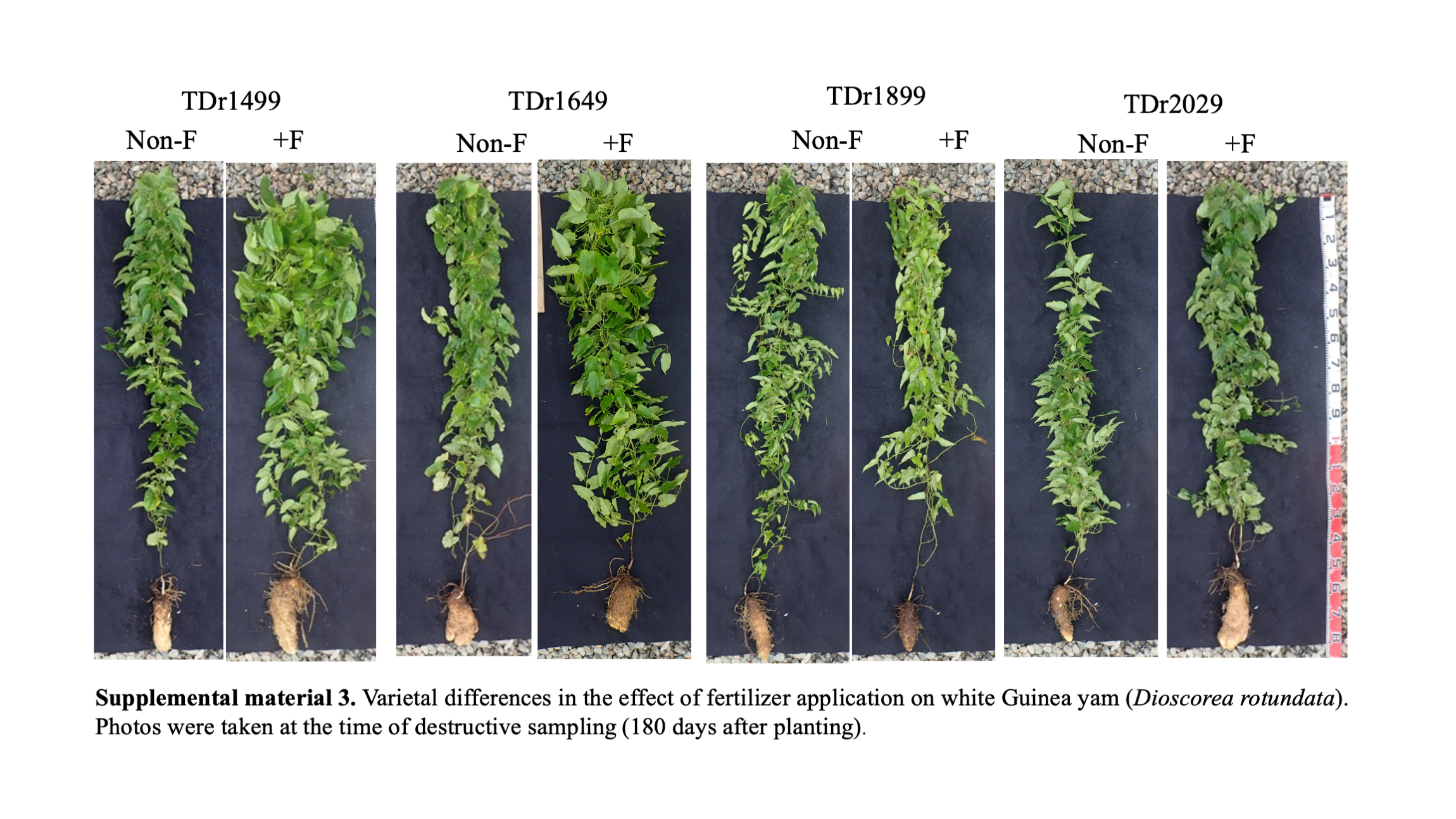

Supplement: Supplementary file 3 [file Image_3.tiff]
